# Two successful spontaneous pregnancies, single and twin, in uterus bicornis unicollis after deep infiltration endometriosis surgery

**DOI:** 10.5935/1518-0557.20200019

**Published:** 2020

**Authors:** Luciene K Tsukuda, Aline R Lorenzon, Tatiana CS Bonetti, Paulo Cesar Serafini, Eduardo LA Motta, Ricardo MA Pereira, Thais Sanches Domingues

**Affiliations:** 1 Huntington Medicina Reprodutiva, São Paulo, SP, Brasil; 2 Departamento de Ginecologia - Escola Paulista de Medicina - Universidade Federal de São Paulo, São Paulo, SP, Brasil; 3 Departamento de Obstetrícia e Ginecologia - Faculdade de Medicina - Universidade de São Paulo, São Paulo, SP, Brasil; 4 Centro de Endometriose - Centro de Reprodução Humana Santa Joana - Hospital e Maternidade Santa Joana, São Paulo, SP, Brasil

**Keywords:** uterus bicornis unicollis, deep infiltrating endometriosis surgery, infertility, spontaneous conception.

## Abstract

A 26-year-old patient was admitted in our center with one year of infertility history after a miscarriage. She was diagnosed with uterus bicornis unicollis and deep infiltrating endometriosis (DIE); therefore, she underwent endometriosis focus removal surgery. After six-months, she conceived spontaneously and delivered one healthy baby. One year after the first pregnancy delivery, she conceived spontaneously and delivered twins in an extremely rare condition of uterus bicornis unicollis, of which there are only 15 cases reported worldwide. Both pregnancies were monitored every two or three weeks using ultrasonography to assess fetal growth, and cervical length was measured to assess the risk of premature delivery.

## INTRODUCTION

Uterine congenital malformations are the most common congenital female genital tract abnormalities (Doruk *et al*., 2013). The incidence is 25% in women with late first or second trimester pregnancy loss or preterm delivery (Doruk *et al*., 2013). Bicornuate uterus results from incomplete fusion of the Mullerian tubes or failure of septum resorption (Arora *et al*., 2007).

Deep infiltrating endometriosis (DIE) is characterized by the presence of glandular and stromal endometrial tissue multifocal lesions outside the endometrium cavity. These lesions extend 5 mm from the peritoneal surface, embrace adjacent structures, and are generally associated with fibrotic reactions. These patients generally report dysmenorrhea, dyspareunia, dyschezia, and infertility (Chamié *et al*., 2010).

We report an extremely rare case of spontaneous single pregnancy followed by twin pregnancy in a woman with uterus bicornis unicollis after DIE surgery.

## CASE REPORT

**Figure 1:**
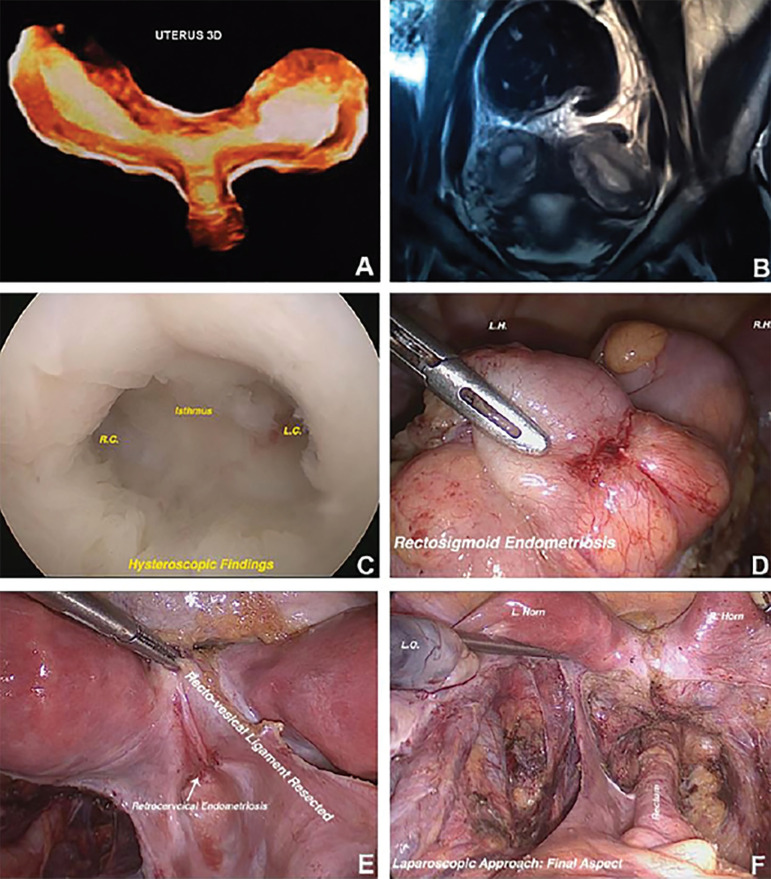
A) View of uterus in 3D ultrasound. B) View of uterus in magnetic resonance imaging (MRI). C) Hysteroscopic view of uterus. D) Rectosigmoid endometriosis. E) Recto-vesical ligament resected. F) Final aspect after laparoscopic approach. RC: Right Horn; LR: Left Horn; LO: Left Ovary

A 26-year-old woman was admitted in our unit in 2013 with one year of secondary infertility history after one spontaneous miscarriage. Uterus bicornis unicollis was diagnosed in the initial evaluation using bidimensional ultrasound (US 2D) complemented with tridimensional (3D) and magnetic resonance imaging (MRI) (Figure 1A and B). Transvaginal ultrasound after bowel preparation for DIE was performed and confirmed retrocervical, uterosacral ligaments and sigmoid colon thickening. She underwent hysteroscopy and laparoscopy posteriorly for endometriosis focus removal (Figure 1 C-F).

The couple entered a timed intercourse program in 2014 and had a single pregnancy in the right horn 6 months after DIE surgery. Pregnancy was monitored via ultrasound examination every two to three weeks, and cerclage was performed at 13 weeks. Low segmental Caesarean section was performed at 34 weeks and 5 days due premature membrane rupture. A baby girl was born healthy at 2909 g, and Apgar scores were 8 at 1 min and 9 at 5 min. Mother and baby were discharged from the hospital three days after delivery.

**Figure 2:**
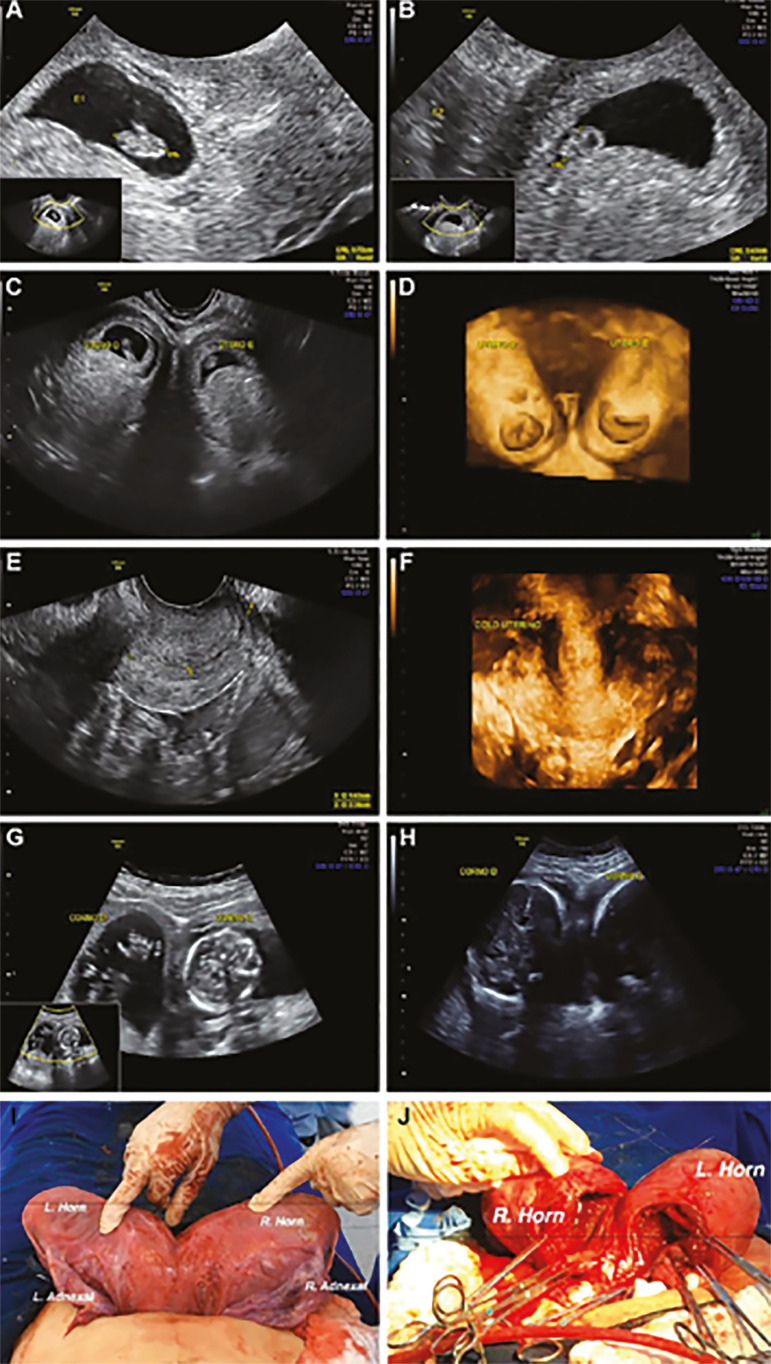
A) Embryo measuring 7.2 mm. B) Embryo measuring 4.7 mm. C) Both embryos, one in each horn, in 2D imaging. D) Both embryos, one in each horn, in 3D imaging. E) Cervix length in 2D. F) Cervix length in 3D. G) Ultrasound image at 16 weeks. H) Ultrasound image at 31 weeks. I) Uterus bicornis unicollis exposed. J) Uterus bicornis unicollis after c-section. Utero D: Right uterus; Utero E: Left uterus; Colo Uterino: Cervix Length; Corno direito: Right Horn; Corno esquerdo: Left Horn. R: Right; L: Left

A spontaneous twin pregnancy occurred in 2016 (29 years old) one year after delivery. Ultrasound scan showed two live embryos, and both gestational sacs were located in separate uterine cavities at 6 weeks and 3 days. The embryos measured 7.2 mm and 4.7 mm (Figure 2A, B and C). A 3D ultrasound exam using a GE Voluson S6 with transvaginal probe was performed and showed one twin in each horn (Figure 2D).

The pregnancy was examined regularly every 3 weeks. Screening of the first trimester pregnancy using nuchal translucency was performed, and each fetus was normal. The pregnancy followed without complications. Cervix length measured 38 mm, and cerclage was not needed or performed. (Figure 2E and F). The patient was followed weekly after 32 weeks of pregnancy, and both fetuses grew properly on Doppler studies. The male fetus was in the right horn, and a female fetus was in the left horn (Figure 2G and H).

At 37 weeks of pregnancy, she was admitted to the hospital with uterine contractions, and two separate lower segment Cesarean sections (LSCS) were performed (Figure 2I and J). Both twins were delivered successfully with birth weights of 2.440 g (female) and 2.850 g (male). Apgar scores were 9 at 1 min and 10 at 5 min for both newborns. The patient and the babies were discharged from hospital three days after delivery. There were no complications in the early or late postoperative periods.

## DISCUSSION

The management of endometriosis-related infertility remains controversial, and optimal care should be individualized (Guinard *et al*., 2017; American Society for Reproductive Medicine - ASRM, 2012). A lack of consensus remains because different types of treatment strategies lead to similar pregnancy outcomes. The most commonly used treatments are videolaparoscopy and *in vitro* fertilization, and generally the woman’s age, time of infertility, and semen analysis helps choose the treatment that will likely lead to better results (Guinard *et al*., 2017). However, recent studies demonstrated that surgery improved pregnancy rates in infertile women diagnosed with endometriosis, mostly in younger women (Bianchi *et al*., 2009).

A twin pregnancy with one gestational sac located in each horn of a uterus bicornis unicollis is an extremely rare condition, especially if the pregnancy is conceived spontaneously. We understood that it is a potentially high-risk pregnancy, and a literature review showed only 15 cases of twin pregnancies associated with a bicornuate uterus (Arora *et al*., 2007; Cruceyra *et al*., 2011; Doruk *et al*., 2013; Nappi *et al*., 2015).

Optimal twin pregnancy follow up is controversial in patients with Müllerian fusion anomalies, and treatment must be individualized because of the risks and rare occurrence of these cases. Serial ultrasound every two or three weeks is a worthy option to assess fetal growth and measure cervical length to assess the risk of premature delivery.
